# Low-volume resuscitation with normal saline is associated with microvascular endothelial dysfunction after hemorrhage in rats, compared to colloids and balanced crystalloids

**DOI:** 10.1186/s13054-017-1745-7

**Published:** 2017-06-29

**Authors:** Luciana N. Torres, Kevin K. Chung, Christi L. Salgado, Michael A. Dubick, Ivo P. Torres Filho

**Affiliations:** 1 Damage Control Resuscitation, US Army Institute of Surgical Research, JBSA Fort Sam Houston, San Antonio, TX USA; 2 Brooke Army Medical Center, JBSA Fort Sam Houston, San Antonio, TX USA; 3 Uniformed Services University of the Health Sciences, Bethesda, MD USA

**Keywords:** Critical care, Microcirculation, Permissive hypotension, Leukocyte, Barrier function, Permeability

## Abstract

**Background:**

Restoration of endothelial glycocalyx (EG) barrier may be an essential therapeutic target for successful resuscitation. The aim of this study was to compare in vivo the effects of resuscitation with normal saline (NS) to lactated Ringer’s solution (LR), 5% albumin and fresh frozen plasma (FFP) on their ability to maintain EG and barrier function integrity, mitigate endothelial injury and inflammation, and restore vascular homeostasis after hemorrhagic shock.

**Methods:**

Anesthetized rats (N = 36) were subjected to hemorrhagic shock (bled 40% of total blood volume), followed by resuscitation with 45 ml/kg NS or LR, or 15 ml/kg 5% albumin or FFP. Microhemodynamics, EG thickness, permeability, leukocyte rolling and adhesion were assessed in >180 vessels from cremaster muscle, as well as systemic measures.

**Results:**

After hypotensive resuscitation, arterial pressure was 25% lower than baseline in all cohorts. Unlike FFP, resuscitation with crystalloids failed to restore EG thickness to baseline post shock and shedding of glycocalyx proteoglycan was significantly higher after NS. NS decreased blood flow and shear, and markedly increased permeability and leukocyte rolling/adhesion. In contrast, LR had lesser effects on increased permeability and leukocyte rolling. Albumin stabilized permeability and white blood cell (WBC) rolling/adhesion post shock, comparable to FFP.

**Conclusions:**

Resuscitation with NS failed to inhibit syndecan-1 shedding and to repair the EG, which led to loss of endothelial barrier function (edema), decline in tissue perfusion and pronounced leukocyte rolling and adhesion. Detrimental effects of NS on endothelial and microvascular stabilization post shock may provide a pathophysiological basis to understand and prevent morbidity associated with iatrogenic resuscitation after hemorrhagic shock.

## Background

Aggressively replacing severe blood loss with unbalanced crystalloids can result in hemodilution, changes in oxygen delivery, iatrogenic acidosis, and coagulopathy [1, 2]. Recently, the concept of damage control resuscitation has emphasized decreased excessive crystalloid volumes and judicious administration of blood products [3]. That has, in turn, resulted in less iatrogenic resuscitation injury, compartment syndromes, multiple organ failure and mortality [3–5]. Resuscitation with plasma has been shown to minimize edema in vitro [6] and improve clinical outcomes [7]. Still, resuscitation with a volume expander may be required for significant hypotension after trauma when blood products are not immediately available [8, 9].

Normal saline (NS) and lactated Ringer’s solution (LR) are commonly used isotonic crystalloid solutions in pre-hospital settings and emergency departments [10]. Of these, NS is the most commonly used solution globally [11]. Although the term “normal saline” was coined because its concentration is 0.9% w/v and 308 mOsm/L, its composition is beyond normal with equal amounts of Na^+^ and Cl^−^, making it both hypernatremic and hyperchloremic relative to the plasma [12, 13]. On the other hand, LR is relatively hypotonic to blood with lower concentrations of Na^+^ and Cl^-^ than NS [1, 11]. Accumulating evidence has implicated intravenous use of NS directly with hyperchloremic metabolic acidosis [1, 10, 11, 14]. Yet, the clinical significance of hyperchloremia remains unclear. Clinical and experimental studies have linked potential hyperchloremia and dysfunction of renal blood flow regulation, even acute kidney injury (AKI) [15–18], but there is no conclusive clinical evidence for the deterioration of renal function secondary to hyperchloremic acidosis induced by NS administration [1, 10, 11, 14, 19, 20].

Adding to the morbidity associated with shock, resuscitation fluids can lead to cardiac and pulmonary complications, systemic inflammatory response, edema, and coagulation and electrolyte/acid-base abnormalities [21]. Associated endothelial cell dysfunction plays a major role in the microcirculation, including leukocyte adhesion to the endothelium, red blood cell (RBC) rheological disturbances, and vascular smooth muscle cell changes, among others. Leukocyte firm adhesion, mediated by endothelial surface adhesion molecules, enables transmigration of leukocytes across the endothelial wall. Inadequate resolution of inflammatory activation due to endothelial dysfunction may augment and prolong the inflammatory process. The endothelial glycocalyx (EG) has become an important focus for control of tissue perfusion, inflammation and transcapillary flow [22–24]. Composed of proteoglycans (syndecan-1) and glycosaminoglycans (heparan sulfate, chondroitin sulfate, and hyaluronan), the EG barrier may be a major determining factor in vascular homeostasis. Thus, degradation of its components has been correlated with other types of endothelial damage and tissue dysfunction in animals and mortality in trauma patients [7, 25, 26].

In recent years, the use of NS in the critically ill has become controversial [10]. Its impact on the vascular endothelium has not been fully elucidated while direct comparisons to other resuscitative fluids are lacking [11, 27, 28]. To fill this gap, the aim of this study was to assess in vivo the endothelial, microcirculatory and systemic impact of NS compared to other commonly available resuscitative fluids. Previously, we have shown that hemorrhagic shock alone in rats induced significant shedding of proteoglycans and marked reduction in EG thickness, exposing the underlying endothelium [24]. We hypothesized that resuscitation with NS potentiates endothelial injury caused by shock and results in significant iatrogenic injury when compared to other fluid options in a rodent model of hemorrhagic shock.

## Methods

This study was conducted in a facility accredited by the Association for the Assessment and Accreditation of Laboratory Animal Care, International. The protocol was approved by our Institutional Animal Care and Use Committee and performed in compliance with the Animal Welfare Act, the implementing Animal Welfare Regulations, and the principles of the Guide for the Care and Use of Laboratory Animals. Male Sprague-Dawley rats (220 ± 10 g body weight) from Charles River Laboratories, Wilmington, MA, USA, breathing spontaneously 100% oxygen, were anesthetized with isoflurane (1.5%) and then tracheostomized to ensure a patent airway.

### Systemic measurements

Arterial and venous catheters (PE 50, Instech Laboratories, Inc., Plymouth Meeting, PA, USA) were inserted for monitoring blood pressure, blood collection and infusion of dyes and other fluids, as described [23, 24]. Arterial blood samples were collected to measure hematocrit, pH, lactate, base excess (BE), creatinine, blood urea nitrogen (BUN), Na^+^, Cl^-^, and K^+^ (I-stat, Abbott, Chicago, IL, USA), syndecan-1, and viscosity. Plasma syndecan-1 was analyzed using a commercial ELISA kit for rats (Antibodies Online, Atlanta, GA, USA). Whole blood viscosity was measured at 37 °C and a shear rate of 225 sec^-1^ using a cone-plate viscometer (Brookfield LV, Middleboro, MA, USA). Total plasma protein was measured using a clinical refractometer (model 300005, Sper Scientific, Scottsdale, AZ, USA). Hemoglobin O_2_ saturation, and respiratory rate were continuously recorded (MouseOx, Starr Lifesciences, Oakmont, PA, USA), along with arterial pressure and heart rate using a data acquisition system (MP150, Biopac, Goleta, CA, USA).

### Skeletal muscle preparation

The cremaster muscle was exteriorized, bathed by Krebs-Henseleit solution at 37 °C and pH 7.4 and positioned flat over a thermostatically controlled pedestal [29]. At the end of surgery, the preparation was covered with a thin impermeable plastic film to minimize dehydration and gas exchange with the atmosphere.

### Description of intravital microscopy setup

The intravital microscopy setup included a microscope (model BX51WI, Olympus America, Center Valley, PA, USA), an immersion objective (Olympus X60, numerical aperture 1.00), a tungsten-halogen lamp for transillumination, a metal halide bulb for epi-illumination, and selective filter block systems for Texas Red (TR) and fluorescein isothiocyanate (FITC) fluorescence. One optical exit of the microscope was connected to a Doppler velocity measuring device (Optical Doppler Velocimeter, Texas A&M, College Station, TX, USA), a color camera (KP-D531U-S3, Hitachi, Woodbury, NY, USA) and a monitor. The second exit was connected to a digital charge-coupled device (CCD) camera (CoolSnap cf; Roper Scientific, NJ, USA) with 1392 × 1040 pixels resolution. The images were digitally stored for later analysis.

### Microhemodynamics assessment

Microhemodynamics assessment included direct measurements of luminal vessel diameter and RBC velocity, assessment of blood flow, wall shear rate (WSR) and wall shear stress (WSS). Luminal diameter was measured using Image-Pro Plus software (MediaCybernetics, Rockville, MD, USA). RBC velocity was determined online using a Doppler device, while blood flow and WSR were calculated from RBC velocity and vessel diameter [30]. WSR was expressed as:

$$ W S R=8\bullet V\bullet D $$

where *V* is the RBC velocity and *D* is the luminal diameter. WSS was defined as:

$$ W S S= W S R\bullet \eta $$

where ɳ is the microvascular blood viscosity.

### Glycocalyx thickness measurements

The EG thickness was estimated using a technique combining the dye-exclusion method [31] with meticulous digital analysis [32], as we have previously described [33]. Dextrans labeled with either TR or FITC were injected at baseline and post-resuscitation, respectively. Briefly, fluorescent and bright-field images were used to measure the width of the fluorescent column and the vessel diameter. All image processing and measurements were performed using Image-Pro Plus software and a high-resolution monitor (Dell Computer Corp., Round Rock, TX, USA) at approximately × 2500 final magnification.

### Microvascular permeability measurements

Using a digital image of the fluorescent post-capillary venule, light intensity was measured in three areas of equal size within the venule (intravascular intensity (Iv)) and three separate areas of equal size in the perivascular space (perivascular intensity (Ip)) for the ratio, in the post-resuscitation period. Using the means of the Ip and Iv measurements, post-resuscitation permeability (vascular leakage) was determined by permeability index (PI), where *PI = Ip/Iv*, as described previously [23]. Permeability indexes only before and after FITC-labeled BSA (FITC-BSA, 10 mg/ml) and data are shown as a ratio relative to the pre-FITC-BSA index (Fig. 2). A ratio of 1.0 denotes no change in vascular leakage after resuscitation, while higher ratios are indicative of changes proportionally higher than the pre-FITC-BSA index (i.e., 1.5 means a 50% increase in vascular leakage, after resuscitation).

### Leukocyte-endothelium interactions in post-capillary venules

Live microscopic images of leukocyte-endothelium interactions in non-branching post-capillary venules were captured in 2-minute digital video recording segments for subsequent analysis, as previously described [34]. Leukocytes were considered adherent to the endothelium if they remained stationary for over 30 s. The number of rolling and/or firmly adherent leukocytes was counted by focusing the microscope objective above and below the diametral plane and was expressed by the number of rolling or adherent leukocytes in 100-μm-length venules, respectively.

### Fluid resuscitation and experimental protocol

For this study, LR was defined in this context as a balanced crystalloid, compared to NS (unbalanced crystalloid). Blood product refers to 5% albumin and FFP. A dose of 15 ml/kg (body weight) is just about the equivalent fluid available for resuscitation of a casualty on the battlefield. FFP, defined as plasma frozen within 6–8 h of collection, was obtained from whole blood from donor rats, collected in sterile syringes with 3.2% citrate and stored at -80 °C or lower, for up to one year. FFP data from a previous study [24] were included in these analyses for use as initial resuscitation by the military. Also, we included 5% albumin, as it is the primary colloid in plasma.

Initially, 5–7 microscopic fields containing post-capillary venules were randomly selected and recorded at baseline, as described previously [35]. After 1 h of baseline measurements (time (T)0–T60), a fixed-volume hemorrhage (standardized to animal weight) to a target of 40% of total blood volume (assumed as 6% of body weight) was initiated and carried out for 30 minutes (T60–T90), followed by an additional 30-minute shock period (T90–T120). Rats were randomized into four resuscitation groups (in T120–T180): (1) NS; (2) LR; (3) 5% albumin; and (4) FFP. In an attempt to obtain similar plasma expansion, the dose for crystalloids was 45 ml/kg at an infusion rate of 1.5 ml/kg/min, and 15 ml/kg at 1.0 ml/kg/min for blood products. Two sets of systemic parameters were collected coinciding with the microcirculatory data, during baseline (T0–T60) and post-resuscitation (>T180). Sham-treated rats were subject to all procedures except induction of hemorrhage or resuscitation.

### Statistical analysis

Statistical analyses were performed using SigmaPlot 12 (Systat Software, Inc., San Jose, CA, USA). Deviation of systemic and microvascular data from the Gaussian distribution was tested using the Shapiro-Wilk test. Two-way repeated measures analysis of variance (ANOVA) was conducted on parametric data and all pairwise multiple comparisons were corrected using the Bonferroni method, if the overall ANOVA was significant (*p* < 0.05). If data were non-normally distributed, Kruskal-Wallis one-way ANOVA on ranks was used, followed by Dunn’s test for all pairwise multiple comparisons (among the groups), when ANOVA was significant. Values are reported as either mean ± SD or median and interquartile range (IQR), as appropriate.

## Results

Sham-treated animals (N = 7 rats) were systemically stable throughout the experimental period (time control). Animals that had induction of hemorrhage (N = 36 rats) were bled on average at a rate of 23.9 ± 0.1 ml/kg. The respiratory rate was not different among all the groups at baseline (64 ± 7 min^-1^) and remained similar after hemorrhage (70 ± 7 min^-1^). Systemic hemodynamic and laboratory parameters are presented in detail in Table 1. Results on shock are presented as single averages for each variable, as the results during shock were not statistically different among groups (except for sham). After limited resuscitation, MAP was higher than in the shock period, but only about 70% compared to sham in all groups (*p* < 0.05). Hemorrhage significantly compromised acid-base balance and tissue perfusion (pH, lactate and base excess) compared to sham (*p* < 0.05), consistent with a shock state, which was corrected by all resuscitation strategies. While all resuscitative strategies reduced hematocrit and blood viscosity, only NS significantly decreased the strong ion difference (SID) and increased Cl^-^ and K^+^. Creatinine and BUN were highest in the NS group compared to sham and other fluids.

**Table 1:** Systemic hemodynamics and laboratory parameters for in vivo blood measurements made in Sham rats as well as in rats subjected to hemorrhage (Shock) followed by resuscitation treatment with normal saline, lactated Ringer’s solution, 5% albumin and fresh frozen plasma (Post-resuscitation)

	Sham		Normal saline	Lactated Ringer’s	5% Albumin	Fresh frozen plasma
Parameters		Shock	Post res	Post res	Post res	Post res
Mean arterial pressure (mmHg)	100 ± 9	46 ± 6^a^	78 ± 12^a,e^	78 ± 10^a,e^	77 ± 9^a,e^	81 ± 9^a,e^
Heart rate (bpm)	371 ± 27	261 ± 30^a^	352 ± 30^e^	369 ± 24^e^	389 ± 10^e^	365 ± 44^e^
PaO_2_ (mmHg)	401 ± 60	224 ± 76^a^	383 ± 33^e^	399 ± 68^e^	377 ± 78^e^	343 ± 57^e^
Arterial pH	7.425 ± 0.028	7.049 ± 0.153^a^	7.416 ± 0.032^e^	7.434 ± 0.036^e^	7.426 ± 0.038^a,e^	7.463 ± 0.080^a,e^
Lactate (mmol/l)	1.5 (1.2–2.0)	4.9 (4.4–5.9)^a^	1.5 (1.1–1.8)	1.2 (1.1–2.5)	1.2 (0.9–1.3)	1.3 (1.1–1.6)
Base excess (mmol/l)	3.7 ± 3.0	-11.8 ± 3.6^a^	2.9 ± 1.5^e^	5.0 ± 3.7^e^	6.9 ± 1.6^e^	3.8 ± 2.4^e^
K^+^ (mmol/l)	4.1 ± 0.4	--	5.5 ± 0.4^a^	5.2 ± 0.9^a^	4.7 ± 0.9^a^	4.8 ± 0.8
Na^+^ (mmol/l)	135.0 ± 1.6	--	138.8 ± 2.0^a^	135.6 ± 1.0	135.9 ± 1.5	136.0 ± 1.0
Cl^-^ (mmol/l)	101 ± 2	--	108 ± 3^a,b,c,d^	103 ± 4^a^	101 ± 4	103 ± 2
Strong ion difference	38.4 ± 0.9	--	35.5 ± 2.1^a,b^	37.8 ± 3.2	40.3 ± 2.9	38.0 ± 2.0
Hematocrit (%)	40 ± 3.0	--	26.2 ± 2.0^a^	25.2 ± 4.0^a^	22.9 ± 1.9^a^	26.3 ± 1.2^a^
Plasma protein (g/dl)	4.2 ± 0.2	--	3.2 ± 0.3^a,b,c^	3.4 ± 0.2^a,b,c^	4.0 ± 0.1	4.2 ± 0.1
Blood viscosity (cP)	4.1 ± 0.4	--	2.6 ± 0.3^a,b,c^	2.4 ± 0.1^a,b,c^	3.5 ± 0.3^a^	4.1 ± 0.3
Creatinine (mg/dl)	0.2 ± 0.1	--	0.6 ± 0.1^a,b,c,d^	0.4 ± 0.1	0.5 ± 0.1^a^	0.4 ± 0.1
BUN (mg/dl)	24.9 ± 0.5	--	40.3 ± 2.3^a,b.c,d^	35.0 ± 5.0^a^	32.5 ± 3.9^a^	37.8 ± 4.9^a^

Values expressed as mean ± SD, except for lactate (median (IQR)). *PaO*_*2*_ arterial partial pressure of oxygen, *BUN* blood urea nitrogen, *Post res* post resuscitation. ^a^Significantly different from sham. ^b^Significantly different from 5% albumin group. ^c^Significantly different from the fresh frozen plasma group. ^d^Significantly different from the lactated Ringer’s group. ^e^Significantly different from the shock period

Microhemodynamics in NS-treated rats indicated that venular RBC velocity, blood flow, WSR and WSS were lower than at baseline and lower than in albumin-treated rats (*p* < 0.05), but similar to results obtained with LR (Table 2). In contrast, albumin or FFP improved microhemodynamics compared to NS (*p* < 0.05).

**Table 2:** Microhemodynamics parameters before (baseline) and after hemorrhage followed by resuscitation treatment with normal saline, lactated Ringer’s solution, 5% albumin and fresh frozen plasma (post-resuscitation)

	Normal saline (n = 48)		Lactated Ringer’s solution (n = 34)		5% Albumin (n = 57)		Fresh frozen plasma (n = 38)	
Parameters	Baseline	Post res	Baseline	Post res	Baseline	Post res	Baseline	Post Res
Venular diameter (μm)	16.6 (14.4–18.8]	16.0 (13.0–18.6)	16.6 (14.9–18.2)	16.0 (13.0–17.3)	15.5 (12.7–17.9)	14.4 (11.2–17.5)	15.4 (14.0–17.7)	14.6 (13.1–18.0)
RBC velocity (mm/s)	1.19 (0.85–2.04)	0.60 (0.30–1.19)^a,b^	1.42 (0.90–2.59)	0.78 (0.46–2.13)	1.37 (1.01–2.24)	0.92 (0.64–1.60)	1.28 (0.89–2.04)	0.82 (0.44–1.44)
Blood flow (×10^-4^ mm^3^/s)	1.43 (0.88–2.67)	0.73 (0.23–1.98)^a^	1.65 (1.02–3.21)	0.91 (0.49–2.31)^a^	1.51 (0.85–2.62)	0.86 (0.51–1.89)	1.59 (1.01–2.17)	0.82 (0.45–1.32)
Wall shear rate (× 10^3^ s^-1^)	0.64 (0.35–1.96)	0.30 (0.17–0.51)^a,b^	0.69 (0.47–1.08)	0.49 (0.24–1.00)^a^	0.82 (0.42–1.20)	0.57 (0.38–0.85)	0.78 (0.42–1.11)	0.43 (0.19–0.86)
Wall shear stress (dyn/cm^2^)	3.20 (1.63–4.70)	0.83 (0.44–1.55)^a,b,c^	3.28 (2.46–4.50)	1.16 (0.56–2.33)^a^	3.17 (1.92–5.41)	1.88 (1.11–2.99)^a^	3.06 (2.04–4.08)	1.53 (0.77–4.06)^a^

Values expressed as median (IQR) for all parameters. *RBC* red blood cell, *Post res* post resuscitation. ^a^Significantly different from baseline. ^b^Significantly different from the 5% albumin group. ^c^Significantly different from the fresh frozen plasma group

**Fig. 1:**
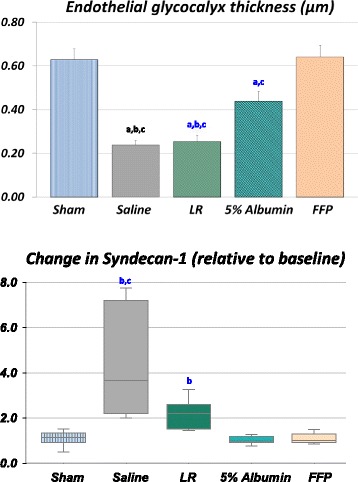
Endothelial glycocalyx thickness (*top*) in post-capillary venules and plasma syndecan-1 after shock followed by resuscitation with normal saline (9 rats, 48 vessels), lactated Ringer’s solution (*LR*) (7 rats, 34 vessels), 5% albumin (12 rats, 57 vessels) or fresh frozen plasma (*FFP*) (8 rats, 38 vessels). The sham-procedure group (time control) was not subjected to hemorrhage or resuscitation (7 rats, 29 vessels). Thickness was taken as the distance between the edge of the dextran column and the surface of the endothelium. In all the groups, resuscitation led to shedding of glycocalyx (*p* < 0.05), but glycocalyx thickness after saline was significantly lower than in the sham, 5% albumin and FFP groups (*p* < 0.05). Similarly, syndecan-1 levels were significantly higher with crystalloid resuscitation compared to colloids. Data for EG thickness are expressed as mean ± SD. Box plots for syndecan display median, interquartile ranges, minimum and maximum. ^a^Significantly different from the sham group. ^b^Significantly different from the 5% albumin group. ^c^Significantly different from the FFP group

Figure 1 (top) shows the effect of different fluids on EG thickness after shock. No resuscitation fluid was able to repair the EG thickness compared to sham (0.61 ± 0.23 μm), except FFP (0.55 ± 0.33 to 0.64 ± 0.32 μm). Although EG thickness was not fully restored in the albumin group, thickness was restored to 81 ± 31% of the baseline (0.55 ± 0.31 to 0.44 ± 0.31 μm), rather than 42 ± 21% and 42 ± 23% observed in the NS group (from 0.61 ± 0.23 to 0.24 ± 0.14 μm) or LR group (from 0.63 ± 0.27 to 0.25 ± 0.16 μm), respectively. Similar conclusions can be drawn from plasma syndecan-1 in Fig. 1 (bottom). The highest shedding in syndecan-1 ectodamains from the endothelial surface was found after NS administration, compared to those levels found in the albumin and FFP groups, respectively. Plasma syndecan was also significantly higher in the LR group, compared to albumin and FFP groups (*p* < 0.05).

**Fig. 2:**
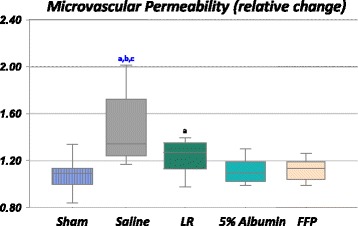
Change in microvascular permeability in rats resuscitated with normal saline (NS) (7 rats, 39 vessels), lactated Ringer’s solution (*LR*) (5 rats, 20 vessels), 5% albumin (6 rats, 32 vessels) or fresh frozen plasma (*FFP*) (7 rats, 28 vessels). Sham-procedure rats (time control) were not subjected to hemorrhage or resuscitation (6 rats, 25 vessels). The permeability index was calculated before and after injection of fluorescein isothiocyanate-bovine serum albumin (FITC-BSA), change in permeability was presented relative to the pre-FITC-BSA index. Permeability changes were higher after NS and LR treatments compared to albumin or FFP (*p* < 0.05). Box plots display median, interquartile range, minimum and maximum. ^a^Significantly different from the sham group. ^b^Significantly different from the 5% albumin group. ^c^Significantly different from the FFP group

Venular barrier function, as assessed by permeability, was significantly worse in the NS group compared with sham, albumin and FFP (Fig. 2). Microvascular permeability was significantly higher after shock/resuscitation with NS compared to sham, albumin and FFP (*p* < 0.05), but not LR. Permeability after resuscitation with LR was higher compared to sham only (*p* < 0.05). In contrast, albumin-treated or FFP-treated rats were able to normalize permeability compared to sham.

**Fig. 3:**
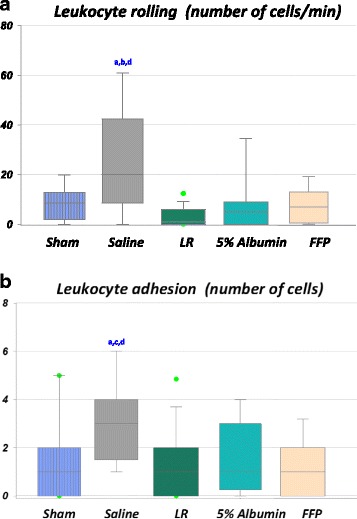
Leukocyte rolling flux (*top*) (**a**) and leukocyte adhesion (*bottom*) (**b**) in post-capillary venules from sham-procedure rats (4 rats, 12 vessels) and from rats subjected to shock/resuscitation with normal saline (NS) (5 rats, 17 vessels), lactated Ringer’s solution (*LR*) (5 rats, 22 vessels), 5% albumin (4 rats, 12 vessels) and fresh frozen plasma (*FFP*) (4 rats, 18 vessels). Significant increase in leukocyte rolling (**a**) and leukocyte adhesion (**b**) after resuscitation with NS compared to sham, LR, albumin and FFP (*p* < 0.05). Leukocytes, stationary for a period of time >30 s were considered firmly adhered to the endothelium surface. The graph shows the number of adherent leukocytes per 100-μm venule length. Box plots display median, interquartile range, minimum and maximum. *Dots* on the *box plots* represent outliers. ^a^Significantly different from the sham group. ^b^Significantly different from the 5% albumin group. ^c^Significantly different from the FFP group. ^d^Significantly different from the LR group

Inflammatory response was assessed by leukocyte rolling and adhesion measurements on the endothelium of post-capillary venules (Fig. 3). Rolling increased after shock/resuscitation with NS compared to LR and albumin (*p* < 0.05, Fig. 3a). Firm adhesion was higher after hemorrhage/resuscitation within the NS group compared to LR, albumin and FFP (*p* < 0.05, Fig. 3b).

## Discussion

The main finding of the present study is that NS prolonged post-shock endothelial injury and caused harmful effects on the microcirculation 1 h after NS administration, compared to LR, albumin and FFP. Saline failed to repair EG, restore syndecan-1 levels, maintain barrier function, improve tissue perfusion, and mitigate leukocyte rolling and adhesion. These findings suggest that NS administration after hemorrhagic shock may have caused endothelial cell dysfunction, which led to propagation of the inflammatory process. Nevertheless, the importance of endothelial modulation and vascular function stability during acidosis and hypovolemic conditions in shock was observed after resuscitation with albumin or FFP by the recovery in the EG and plasma protein and subsequent normalization of permeability, proteoglycans, blood flow increase, and mitigation in inflammatory response.

Animals experienced a significant, yet similar, drop in MAP, heart rate, base deficit and pH, and an increase in plasma lactate levels post-shock due to the development of metabolic acidosis and widespread tissue ischemia/hypoxia. Resuscitation with NS, LR, albumin, and FFP normalized the heart rate and reversed metabolic acidosis, but did not fully restore MAP (permissive hypotension resuscitation protocol). Post-resuscitation SID, plasma Cl^-^ and creatinine were in keeping with clinical studies [15, 17, 36, 37]. Previous studies have shown that pro-inflammatory response and decreased renal blood flow may be due to hyperchloremia [1, 38, 39]. However, lower in-hospital mortality among patients who receive low-chloride intravenous fluids remains controversial [13, 20, 40].

Changes in protein levels of the plasma after resuscitation during hypovolemic shock (colloid-osmotic pressure) may result in modulation of plasma viscosity, intravascular shift of fluids and microhemodyanamics response [24, 41]. A fairly mild alteration in microhemodynamics is linked to clinical signs of impaired perfusion and may be mitigated by optimal fluid therapy, before organ dysfunction. Here, severe hemodilution was present in all groups, but total plasma protein and viscosity were reduced in the NS and LR groups (vs albumin and FFP), in agreement with previously reported clinical studies [42]. Low plasma protein and viscosity may have led to the further decrease in RBC velocity and WSS found in the NS group, but not in the LR group. Similar findings of decreased renal flow velocity after NS administration have been reported in experiments in dogs [18] and in healthy humans, after administration of 30 ml/kg NS [17]. In contrast, animal studies have demonstrated that NS resuscitation in hemorrhagic shock partially restores the renal blood flow [43].

Fluid resuscitation may impact patient morbidity and mortality in the early stages of critical illness and, therefore, should be recommended when physiological goals in the target organ are not met. Here, we demonstrated EG modulation, alteration of the endothelial barrier and aggravated inflammation secondary to hypotensive resuscitation, using crystalloids with low fixed dose (45 ml/kg). Compared to an equal fixed dose of LR, NS had detrimental effects on the microvascular/endothelial outcomes post shock. Whether these outcomes lead to clinical deleterious effects or affect the prognosis of the critically ill patient remains unclear. Major clinical studies that utilized total doses <40 ml/kg (or initial fluid administration of 15–20 ml/kg before randomization) showed similar outcomes between NS and balanced crystalloids for mortality, relative risk of death, acute kidney injury (AKI), or renal replacement therapy [40, 44–46]. On the other hand, clinical studies utilizing total doses >50 ml/kg have emphasized the adverse effects of NS on organ function, mortality, and other clinical outcomes compared to balanced crystalloids [15, 36, 37, 47, 48]. In conclusion, dose-dependency of adverse effects of resuscitation with NS or balanced crystalloids may play a potential role in critically ill patients, and better end-organ physiologic endpoints, such as endothelial biomarkers or microvascular tissue perfusion, are currently lacking [17].

Previous work from our laboratory has suggested that rapid EG shedding in vivo in response to shock may trigger a cascade of signaling events by endothelial cell activation prior to any resuscitation [24, 33]. We have demonstrated that hemorrhagic shock alone caused a significant reduction in EG thickness (0.132 $\pm$ 0.010 μm) in post-capillary venules of cremaster muscle, compared to baseline (0.444 $\pm$ 0.020 μm) [24]. Here, NS and LR administration prolonged EG shedding post shock (0.240 $\pm$ 0.140 μm) compared to that after shock alone. In rats treated with protein-free fluids, i.e., NS or LR, EG thickness remained significantly reduced post shock, possibly due to the loss of EG-adsorbed proteins and proteoglycans. On the other hand, albumin or FFP infusion showed a promising effect by improving EG thickness post shock. Unlike enzymatic digestion of a single glycan in the glycocalyx [49], shedding of EG constituents post shock/trauma after resuscitation with standard-of-care fluids has been described by the outflow of syndecan-1 and other EG components, followed by their increase in plasma [7, 23, 24, 26, 50].

Hemorrhagic shock is a systemic condition of reduced tissue perfusion that compromises the endothelial barrier in several organs. Previously, we reported a significant rise in plasma syndecan-1 secondary to hemorrhagic shock in our rodent model [24]. Our data showed plasma syndecan-1 was highest in NS-resuscitated rats, compared to LR, albumin and FFP, suggestive of continuous shedding of EG components from the endothelial surface, and loss of the EG barrier possibly commenced after severe bleeding. In contrast, resuscitation with 5% albumin prevented plasma syndecan-1 shedding, similar to FFP. Recovery of EG thickness and essential EG components, associated with blood rheological properties (plasma viscosity), may modulate the endothelial cell response by providing ideal mechanical conditions for vasodilation and blood flow increase in hypoxic and hypoperfused tissues. According to the revised Starling model [51], the EG layer also offers an extra physical barrier for endothelial cells as a molecular sieve for plasma proteins that establishes the oncotic force across the transvascular wall. We have previously reported that hemorrhagic shock alone causes significant EG shedding and loss of endothelial barrier and plasma-based resuscitation can rebuild the EG layer and stop the shedding of syndecan and other heparan sulfate proteoglycans, in a similar rodent model of hemorrhagic shock [23, 24]. These findings are in agreement with other studies using plasma resuscitation in hemorrhagic shock [50, 52]. Like FFP, restoration of plasma protein levels after administration of a low volume of 5% albumin may account for the partial EG recovery, superior barrier function, and adequate microhemodynamic response (blood flow, WSR, and WSS).

Experimental studies have proposed a connection between endothelial dysfunction, hypernatremia and loss of the filtration barrier [49, 53, 54]. In vitro and in vivo experiments have suggested that in physiological conditions the presence of aldosterone in hypernatremic circumstances may enhance influx of sodium into endothelial cells (stiffening) followed by removal of EG components and endothelial dysfunction [53, 54]. In addition, van den Hoven and colleagues (2008) demonstrated in the glomerular basement membrane of mice the primary role of glycosaminoglycans in charge-selective filtration of plasma proteins, i.e., loss of glycocalyx integrity may not limit sieving of negatively charged plasma proteins (e.g., albumin) through the basement membrane, and may contribute to proteinuria. Our findings support that hypernatremia secondary to NS administration post shock may lead to loss of or neutralization of EG negative charges due to syndecan shedding and EG degradation, which possibly describes the significant loss of vasoprotective function and the filtration barrier. Although it has not yet been clinically proven, restoration of glycocalyx integrity after shock/resuscitation may play a major role as a filtration barrier in endothelial and epithelial cells.

The present work provides further systemic and microvascular evidence that resuscitation with NS should not be considered clinically equivalent to balanced crystalloids [46] or albumin [40]. Resuscitation with NS worsened inflammation post shock by increase in leukocyte rolling and adhesion to the vascular endothelium, likely due to a widespread EG collapse (increased syndecan-1) and greater binding of leukocytes to endothelial cells [22, 55]. These findings agree with studies linking inflammation and NS treatment in animals [56] and humans [17, 48]. A rise in leukocyte adhesion in venules can also increase resistance to flow in the microcirculation [25], with effects that may accentuate tissue hypoperfusion, acidosis and potentiate the inflammatory response. In contrast, albumin infusion did not trigger leukocyte activation and sustain microvascular flow post shock, similar to FFP resuscitation, as previously reported in a swine model of hemorrhage shock [27].

## Conclusions

This is the first study to highlight post-shock detrimental effects of resuscitation with NS on the vascular endothelium and microcirculation, associated with rheological disturbances and loss of the EG barrier. Resuscitation with NS failed to inhibit syndecan-1 shedding and repair the EG, which led to loss of endothelial barrier function (edema), decline in tissue perfusion, and pronounced leukocyte rolling and adhesion. Although the underlying mechanisms may remain elusive, our findings may shed light on clinically relevant adverse consequences associated with iatrogenic resuscitation after NS administration in hemorrhagic shock, such as exacerbated systemic inflammatory response, compartment syndrome, and multiple organ failure. EG degradation, severe hemodilution and loss of barrier function and plasma proteins were secondary to resuscitation with either NS or LR in severely hemorrhaged rats. Compared to LR, NS also showed hyperchloremia, leukocyte rolling/adhesion and permeability, and sustained low blood flow and shear stress. Conversely, administration of protein-rich solutions, i.e., albumin or FFP, helped to rebuild the EG composition post shock. These results further illustrate the microcirculation and endothelium as essential therapeutic targets in critical illness.

## Abbreviations

AKI: Acute kidney injury
BUN: Blood urea nitrogen
EG: Endothelial glycocalyx
ELISA: Enzyme-linked immunosorbent assay
FFP: Fresh frozen plasma
FITC: Fluorescein isothiocyanate
LR: Lactated Ringer’s
MAP: Mean arterial pressure
NS: Normal saline
RBC: Red blood cell
SID: Strong ion difference
TR: Texas Red
WSR: Wall shear rate
WSS: Wall shear stress
WBC: White blood cell
